# Constraints on eQTL Fine Mapping in the Presence of Multisite Local Regulation of Gene Expression

**DOI:** 10.1534/g3.117.043752

**Published:** 2017-06-08

**Authors:** Biao Zeng, Luke R. Lloyd-Jones, Alexander Holloway, Urko M. Marigorta, Andres Metspalu, Grant W. Montgomery, Tonu Esko, Kenneth L. Brigham, Arshed A. Quyyumi, Youssef Idaghdour, Jian Yang, Peter M. Visscher, Joseph E. Powell, Greg Gibson

**Affiliations:** * School of Biological Sciences and Center for Integrative Genomics, Georgia Institute of Technology, Atlanta, Georgia 30332; † Institute for Molecular Biosciences, University of Queensland, Brisbane, Queensland 4072, Australia; ‡ Estonian Genome Center, University of Tartu, 50090, Estonia; § Department of Medicine, Emory University, Atlanta, Georgia 30329; ** Division of Biology, New York University Abu Dhabi, Saadiyat Island, United Arab Emirates

**Keywords:** fine mapping, linkage disequilibrium, multivariable regression, colocalization, gene regulation

## Abstract

Expression quantitative trait locus (eQTL) detection has emerged as an important tool for unraveling of the relationship between genetic risk factors and disease or clinical phenotypes. Most studies use single marker linear regression to discover primary signals, followed by sequential conditional modeling to detect secondary genetic variants affecting gene expression. However, this approach assumes that functional variants are sparsely distributed and that close linkage between them has little impact on estimation of their precise location and the magnitude of effects. We describe a series of simulation studies designed to evaluate the impact of linkage disequilibrium (LD) on the fine mapping of causal variants with typical eQTL effect sizes. In the presence of multisite regulation, even though between 80 and 90% of modeled eSNPs associate with normally distributed traits, up to 10% of all secondary signals could be statistical artifacts, and at least 5% but up to one-quarter of credible intervals of SNPs within *r*^2^ > 0.8 of the peak may not even include a causal site. The Bayesian methods eCAVIAR and DAP (Deterministic Approximation of Posteriors) provide only modest improvement in resolution. Given the strong empirical evidence that gene expression is commonly regulated by more than one variant, we conclude that the fine mapping of causal variants needs to be adjusted for multisite influences, as conditional estimates can be highly biased by interference among linked sites, but ultimately experimental verification of individual effects is needed. Presumably similar conclusions apply not just to eQTL mapping, but to multisite influences on fine mapping of most types of quantitative trait.

Since it is now recognized that many SNP–trait associations identified by genome-wide association studies (GWAS) can be attributed to effects on gene expression, precise estimation of the location and effect sizes of regulatory polymorphisms has become important for understanding the relationship between genetic and phenotypic variation (Maurano *et al.* 2012; Farh *et al.* 2015). eQTL analysis and related functional genomic strategies are, thus, now a standard component of genetic fine mapping (Nicolae *et al.* 2010). The minimal expectation is that they can identify the gene within a locus that accounts for a GWAS signal, although even this is a far from trivial undertaking (Chung *et al.* 2014; Pickrell 2014). Many investigators make the stronger assumption that colocalization of eSNP and GWAS signals to a tight LD interval implies the ability to define if not the causal variant, then at least a credible set of SNPs that include the causal site (Trynka *et al.* 2013; Gaulton *et al.* 2015; Kichaev and Pasaniuc 2015; Liu *et al.* 2016). Studies across a wide range of organisms including yeast, mice, and several plant species, reviewed by Albert and Kruglyak (2015) and Cubillos *et al.* (2012), show that individual regulatory substitutions can be experimentally defined and linked to visible phenotypes. Similarly, the *SORT1* example in humans (Musunuru *et al.* 2010) showed how dissection of the path from regulatory variant to tissue-specific expression can define causal influences on (heart) disease. However, this is painstaking work that relies on strong prior statistical or functional prediction of likely credible intervals. The enrichment of chromatin marks such as DNAse hypersensitive sites in the vicinity of eQTL validates the assumption that many credible intervals encompass regulatory SNPs (ENCODE Project Consortium 2012; Roadmap Epigenomics Consortium 2015), but conversely raises the question of why there are so many instances of discordant fine localization (Huang *et al.* 2015; Chun *et al.* 2017); does this reflect biochemistry (regulatory sites do not always map to ENCODE elements), or simply limits to the statistical resolution of association signals (Udler *et al.* 2010)?

The general and parsimonious assumption is that functional variants are sparsely distributed, and hence that their precise localization or estimation of effect sizes is not affected by interference due to confounding of statistical signals. However, as GWAS have increased in size, it has become clear that multisite effects are not uncommon. For example, the latest meta-analysis of height suggests that over one-third of the >400 identified loci have multiple independent signals (Wood *et al.* 2014), and that the expression of a large proportion of genes in lymphocyte cell lines is regulated by two or more locally acting variants (*cis*-eQTL) (Liang *et al.* 2013). Since LD within a locus can be extensive, the potential for misestimation of eQTL effects due to interference between signals from tightly linked polymorphisms is high. Here, we address this concern through a combination of simulation studies.

Heritability analyses in recent years have shown that, on average, up to half of the variance of phenotypic traits, or of transcript abundance, can be explained by genetic factors, mostly acting in an additive manner (Powell *et al.* 2013; Wright *et al.* 2014). An important difference with visible phenotypic traits is that one or a few SNPs are often found to explain a large proportion of the genetic variance. These typically lie within 1 Mb of the transcript and are regarded as *cis*-acting regulatory polymorphisms. As shown by Lloyd-Jones *et al.* (2017), 35% of all expressed genes in peripheral blood have narrow sense heritability >0.1, with a median of 0.3. The sentinel *cis*-eQTL, namely the SNP with the strongest association signal at a locus, typically explains 85% of the locally acting variance, which is two-thirds of that attributed to all detected eQTL, but the majority of the genetic variance is generally actually due to *trans*-acting polymorphisms of small effect.

The largest blood eQTL study reported to date, assembled from meta-analysis of over 5000 individual Illumina microarray samples (Westra *et al.* 2013), reports single site local associations that are genome-wide significant for 6418 genes (44% of those tested) with a 5% false discovery rate. However, the blood eQTL browser only provides single site (unconditional) estimates for all local SNPs at each locus. A more powerful cross-population Bayesian method (Gusev *et al.* 2014), applied to just 420 lymphocyte cell lines in the Geuvadis dataset (Lappalainen *et al.* 2013), found a very similar number of genes with evidence for regulation by a local eQTL (eGenes), 14% of which had strong evidence for secondary association signals in a multisite analysis (Wen *et al.* 2015).

While increased sample size is certain to reveal specific instances of multisite regulation, it also provides the opportunity to more accurately define effect sizes in the presence of multiple sites that have varying degrees of LD. Figure 1 illustrates the reasoning for a hypothetical case. Five true eSNP effects are indicated by red lines with increasing effects above the horizontal and decreasing below it, while single site unconditional estimates at common variants across the locus are indicated as black lines. Where two sites in high LD have effects in the opposite direction (green circle), they will either cancel each other out or substantially bias the effect size estimates. Where two sites act in the same direction (blue circle), their effects will tend to be added together, and hence the strongest association will overestimate the effect while the secondary site will be underestimated or not detected. Weaker associations (brown circle) may also go undetected if they are influenced by even low levels of LD. In theory, if the location of the functional sites is known *a priori*, these difficulties can be resolved by multisite linear regression simultaneously fitting all of the identified SNPs. In practice, the identities of the functional sites are unknown, and exhaustive multisite modeling is impractical, so sequential conditional analyses are used to find secondary, tertiary, and so forth associations that are independent of the primary signal. In the presence of strong LD, this approach is expected to miss independent associations, which will remain confounded with the primary signal.

**Figure 1:**
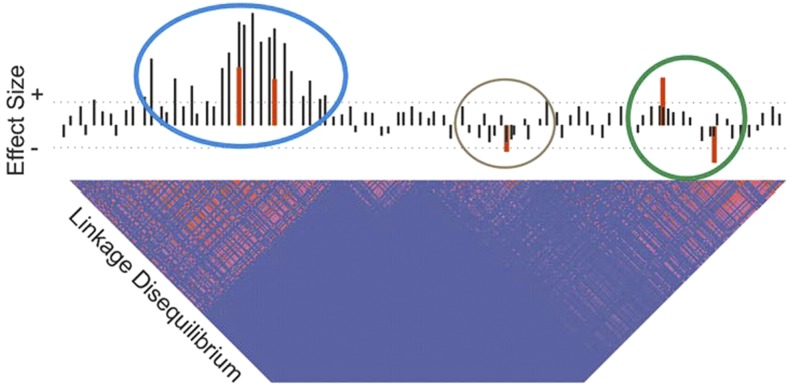
Schematic of multisite regulation of gene expression. Black bars indicate univariate estimates of allelic effects of minor alleles increasing (above the horizontal) or decreasing (below the horizontal) gene expression without conditioning on other sites. Red bars show the actual effects at five single nucleotide polymorphisms in this locus, which has a linkage disequilibrium profile with two large and one small block of elevated linkage disequilibrium (pink squares). Dotted horizontal lines indicate a statistical significance threshold, which is only exceeded in the univariate modeling by the two left-hand sites (blue circle). Since these two sites act in the same direction, they reinforce one another, leading to overestimation of their effect sizes, whereas the two at the right (green circle) interfere with one another antagonistically, leading to underestimation of their effects. The effect at the fifth site (brown circle) may only be identified following conditional analysis.

Several Bayesian methods have recently been introduced that should improve localization of linked causal variants, although they do not in general estimate effect sizes. CAVIAR (Hormozdiari *et al.* 2014) enumerates all possible causal states for one or more sites in a short interval of ≤100 SNPs, but to control the computational burden, the maximum number of causal variants is typically set to two. It is claimed to improve identification of causal variants by 20–50% over existing methods such as BIMBAM (Servin and Stephens 2007). The eCAVIAR extension for combined eQTL and GWAS analysis (Hormozdiari *et al.* 2016) uses a greedy method to find a subset of SNPs with a specific confidence (95% by default) that causal variants are identified as candidates. PAINTOR (Kichaev *et al.* 2014) uses a similar algorithm, whereas FM-QTL (Wen *et al.* 2015) applies an MCMC algorithm to explore the causal status space, utilizing a posterior inclusion probability to choose the causal variant credible interval DAP software was then developed (Wen *et al.* 2016) to explore high probability causal intervals with reasonable runtime. FINEMAP (Benner *et al.* 2016) uses logical schema that is similar to that of CAVIAR, but adopts a Shotgun Stochastic Search method to restrict the search space and focus on combinations of high probability intervals.

This study explores the sources of error in estimating eQTL effects. We start by using simulations to ask how multisite regulation influences (i) the number of independent peaks detected by stepwise conditional analysis, (ii) the accuracy of localization of true causal variants, and (iii) the effect size estimation of discovered causal variants. We also address the question of what proportion of discovered peaks may be driven by undocumented variants in LD with the genotyped sites, and conclude with a comparison of the performance of two recently developed Bayesian joint localization methods, eCAVIAR and DAP, finding minor improvements in detection of linked causal variants but little impact on fine mapping, particularly in regions of high LD or if sample sizes are small.

## Methods

### Consortium for the Architecture of Gene Expression (CAGE) dataset

Our simulations utilize genotypes obtained from the CAGE dataset, which consists of Illumina HT12 microarray-based gene expression profiles, as well as whole-genome genotype information from five research studies: the Brisbane Systems Genetics Study (BSGS, *N* = 926) (Powell *et al.* 2012), the Atlanta-based Centre for Health Discovery and Well-Being (*N* = 439) (Wingo and Gibson 2015) and Emory Cardiology Genebank (*N* = 147, Kim *et al.* 2014), the Estonian Genome Centre, University of Tartu study (*N* = 1065, Schramm *et al.* 2014), and the Morocco Lifestyle study (*N* = 188, Idaghdour *et al.* 2010), for a total of 2765 individuals. Since the BSGS sample includes twins, it was removed to avoid complications of relatedness, leaving a set of 1839 European-ancestry unrelated individuals. IRB approval was obtained for the combination of data into a mega-analysis, both by the University of Queensland and for each participating site.

Genotype imputation for the CAGE cohort was performed jointly on the five contributing studies to ensure uniformity of assignment of strand identities of SNPs, and is described in detail in Lloyd-Jones *et al.* (2017) and at https://github.com/CNSGenomics/impute-pipe. Briefly, the pipeline was as follows: (1) preimputation quality control and data consistency checks; (2) imputation to the 1000G reference panel with Impute2 (Howie *et al.* 2012); (3) postimputation quality control (filtering on various data features); and (4) merging datasets on common SNPs.

### Simulation studies

Four different simulation studies were conducted. In all cases, we use the terminology uni-site (univariable) to refer to models where a single causal variant is modeled as a fixed effect, and multisite (multivariable) where two or more variants are modeled, also as fixed effects. The term multivariate modeling is used for situations where there are two or more dependent variables, whereas in these models we are assessing the joint effects of two or more causal variables. Some models also incorporate random effects of covariates such as a genetic relationship matrix.

The first set of simulations assessed the power and accuracy of two site regressions assuming that the identities of the two causal variants are already known. We modeled the influences of effect size, minor allele frequency (maf), LD, and sample size. Environmental variance was randomly generated as a z-score (mean 0 and SD 1) and genotype effects (β) were added in SD units (sdu) multiplied by ± 0, 1, or 2 according to genotype so as to account for from 2 to 30% of the phenotypic variance, computed as 2*p*(1−*p*)β^2^. Thus, an allele with β = 0.8 is expected to explain 20% of the variance if maf *P* = 0.2, or 32% of the variance if *P* = 0.5. The influence of LD was assessed at *r*^2^ = 0.1, 0.5, or 0.9, noting that as LD increases, high *r*^2^ values are not obtained for combinations of a rare and a common allele. For each combination of parameter values, we generated 1000 randomizations of the environmental variance, and assessed (i) the univariate estimate at each genotype, (ii) the mean conditional estimate of the second SNP, and (iii) the joint effect estimates with both SNPs. From these values, we computed the mean absolute value of the deviation between the observed estimate and the true effect size from the univariate, conditional, and joint (two site) models. The univariate estimates agree extremely well with expectations from the analytical solution described in the *Results*.

The second simulation study asked whether unimputed variants influence the localization of eSNP signals. Since nonimputed SNPs are not present in the CAGE data, we approximated their identities by randomly sampling from a set of CAGE-imputed SNPs weighted to have the same frequency distribution shown in Figure 5C and assigning effect sizes from 2 to 10% of the variance explained for normally distributed pseudogene expression traits using the CAGE (minus BSGS) genotypes. We then removed the SNP and all other SNPs with *r*^2^ > 0.8, and performed stepwise conditional regression, documenting instances of primary and secondary signals at *P* < 10^−5^, as plotted in Figure 5E. The cumulative proportion of spurious secondary signals was computed by summing the detection rate by the size of the maf bin of the unimputed SNPs.

The third set of simulations were performed to evaluate the difference in effect size estimates using the multisite linear regression method for parameter estimation from data representative of the LD structure in the CAGE dataset. For each of 500,000 iterations, four sites were chosen at random from a window extending from 200 kb upstream of the transcription start site to 200 kb downstream of the transcription termination site of a randomly picked gene in the CAGE cohort (excluding the BSGS data, since it includes twins), and assigned an effect size from a uniform distribution of variance explained (VE) relative to environmental noise ranging between 0.02 and 0.1. The effect size β for an allele with maf *p* is computed as √[VE/2*p*(1−*p*)]. Subsequently, each phenotype was simulated as $\overset{4}{\underset{i=1}{\sum }}\beta_{i}\;\times \;geno_{i}$ + *N*(0,1), where β*_i_* is the simulated allelic effect size for a SNP *i*, and *geno_i_* is the dosage of minor allele at the simulated SNP for a given sample. The significance threshold for sequential conditional detection of the variants in a sample of 1839 CAGE individuals was set at *P* < 10^−5^, since simulations indicated a <1% false discovery rate for null variants at this level. We evaluated (i) how many of the four SNPs were significant in sequential conditional modeling, (ii) the mean LD between each SNP and the other three SNPs in the model, (iii) the effect size estimates from the conditional and joint multisite models, (iv) the difference between these two estimates as a function of the mean LD, and (v) the rank of the discovered SNP for each peak eSNP and the modeled sites, which were assumed to be the causal variants for some trait.

The fourth set of simulations was performed to evaluate the influence of two Bayesian methods for fine mapping that is sensitive to the LD structure at a locus. First, eCAVIAR was used to also assess the accuracy of colocalization of eQTL and GWAS signals. Summary statistics were generated for normally distributed traits where either one, two, or three sites chosen at random from contiguous intervals of 100 SNPs in the full sample of 1835 CAGE genotypes were assigned to explain between 2 and 10% of the variance. Effects were assigned in the same direction for each minor allele. Marginal single site estimates were generated by univariable regression, and then eCAVIAR was used to combine the Posterior Probabilities, which were multiplied together to yield the Combined Likelihood Posterior Probabilities (CLPP) with a significance threshold of 0.001 as recommended (Hormozdiari *et al.* 2016). Owing to the high computational burden, only 4000 simulations were performed. GWAS variants are in general unlikely to explain this amount of variance, but the statistical evidence is approximately equivalent to that expected for typical trait associations where a SNP explains ≤0.1% of the variance in a sample of 20,000 individuals. The effect of sample size was evaluated by fitting a single eQTL effect to just 200 individuals in each simulation. Second, the DAP simulations were performed using the adaptive algorithm, which estimates the number of causal variants from the data and also generates a list of possible sites that could explain the effect(s). Again, owing to the high computational burden, only 130 simulations were performed, using the same parameters as for the sequential conditional analyses with four assigned causal variants. A final set of simulations designed only to fine map three causal sites in a single moderate to high LD block extracted contiguous sets of 100 SNPs, and randomly assigned effects only on the condition that three sites selected from the set each had *r*^2^ > 0.3 with one another.

### Data availability

All scripts are available at https://github.com/jxzb1988/Script-for-constraint-paper.

## Results

### Underestimation of allelic effects by sequential conditional analysis

Our basic simulation framework utilizes the current standard mapping approach of sequential conditional analysis, in which the residuals from discovery of each SNP are taken forward as the dependent variable in a new scan for an independent SNP (Yang *et al.* 2012). To explore the performance of this strategy in the context of four causal regulatory variants in the vicinity of a typical gene, 500,000 simulations were carried out by randomly picking four SNPs within 200 kb up- or downstream of the 5′ and 3′ ends of a randomly chosen gene, from the imputed whole-genome genotypes of 1839 unrelated European-ancestry individuals. We assigned each SNP an allelic effect size so as to explain between 2 and 10% of the variance of a trait otherwise uniformly distributed with a mean of 0 and SD of 1. Power to detect individual univariate effects of this magnitude is close to 100% at the significance level *P* < 10^−5^. The sampling was performed across all genes so as to sample from the typical LD structure in the European-ancestry human genome. Furthermore, effects were randomly assigned under three scenarios, with either four positive (4:0), three positive and one negative (3:1), or two positive and two negative (2:2) effects of the minor allele on the trait. For the eQTL detection, once the sequential conditional detection was completed, we determined which of the four causal variants was in high LD (*r*^2^ > 0.8) with one of the discovered sites. If a peak was in high LD with more than one causal variant, it was assumed that it tagged the highest effect site.

Table 1 summarizes the “tagging efficiency,” namely the percent of simulations in which the indicated number of significant independent sites was detected, as well as the proportion of the discovered variants that are in high LD (*r*^2^ > 0.8) with one of the simulated causal variants. Across all three scenarios, at least three independent peaks are detected in ∼90% of the simulations, and at least four independent peaks in two-thirds of the simulations. Notably, in the scenarios where all four minor alleles influence expression in the same direction, 10% of the simulations detected five or more independent peaks, at least one of which must be a spurious association, despite a <1% false discovery rate for univariate associations of the same magnitude. The proportion of credible intervals (*r*^2^ > 0.8 regions around each discovered variant) that contain the actually simulated site ranges between 85 and 90% in each scenario, again indicating relatively poor localization of the causal variant.

**Table 1:** Tagging efficiency of detection of causal variants with *r*^2^ cutoff 0.8

	Scenario (Positive:Negative Effects)*^a^*		
Number of Detected Causal Variants	4:0	3:1	2:2
1	0.6% (0.84)	1.4% (1.00)	1.2% (0.62)
2	5.7% (0.69)	10.4% (0.91)	12.8% (0.86)
3	28.2% (0.78)	24.3% (0.85)	22.5% (0.76)
4	55.5% (0.89)	59.0% (0.92)	60.2% (0.90)
>4	10.0% (0.63)	5.0% (0.66)	3.3% (0.56)

a % indicates the percent of cases with the indicated number of independent discovered variants; numbers in brackets are the proportion of discovered variants that are in linkage disequilibrium (*r*^2^ > 0.8) with one of the simulated causal variants.

Similar results are reported in Table 2 for the reciprocal measure of what fraction of simulated variants is captured by discovered variants. It includes results for simulations with just two or three causal variants, and reports the percentage of cases where the causal variant was within the *r*^2^ > 0.8 credible interval for a discovered peak. Across the sets of 500,000 simulations, at least two variants are detected >85% of the time, but the power to detect all of the multiple eSNPs is a function of the number of sites operating in the same direction. It is highest for the case where the minor alleles for all four variants have effects in the same direction and least where two are in one direction and two in the opposite direction. In the 4:0 scenario, three or more of the four eSNPs are detected three-quarters of the time, whereas this proportion drops toward two-thirds with the simulations for 2:2. No variants are detected in just over 1% of the simulations, and just one variant in ∼6% of them, while 80% of the variants are detected overall. This proportion rises to 90% for the two-variant simulations, illustrating how multisite interactions reduce the discovery of independent eQTL peaks.

**Table 2:** Detected true causal variants in simulations with four, three, or two causal variants

	4 SNP Scenario			3 SNP Scenario		2 SNP Scenario	
Detected True	4:0	3:1	2:2	3:0	2:1	2:0	1:1
0	1.1%	1.2%	1.4%	2.1%	1.9%	5.5%	6.3%
1	5.5%	6.0%	6.0%	12.1%	15.1%	9.4%	9.1%
2	18.4%	22.3%	23.7%	17.6%	17.9%	85.1%	84.6%
3	23.7%	23.4%	22.1%	68.2%	65.0%		
4	51.3%	47.1%	46.7%				

**Figure 2:**
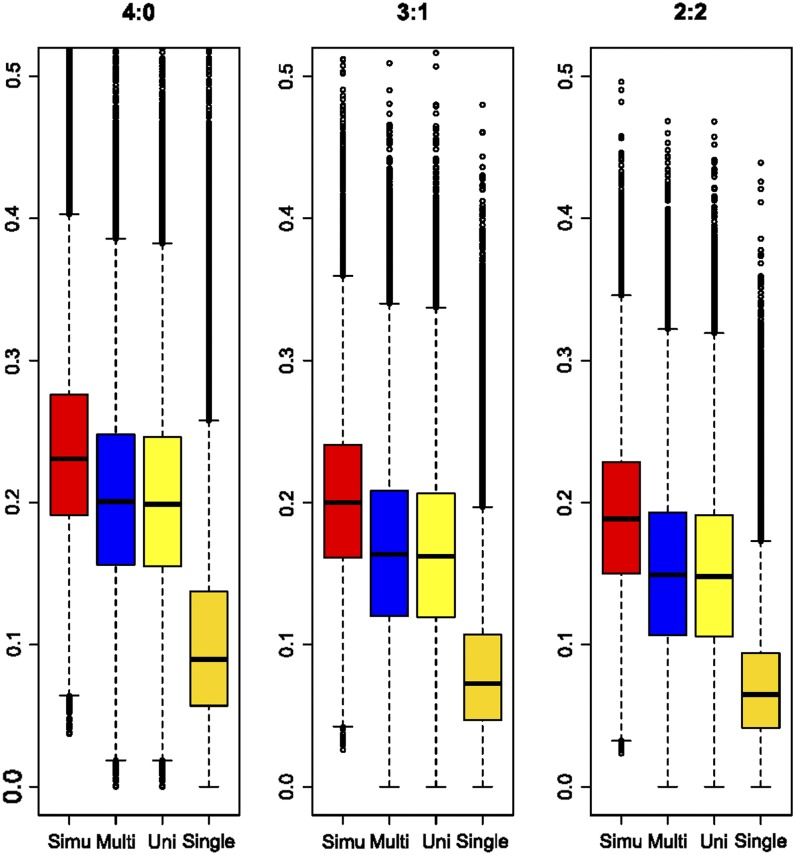
Proportion of variance explained by detected eSNPs in simulations. Box and whiskers show median, interquartile range, and 95% C.I. for the proportion of variance explained under three scenarios for 500,000 simulations of four sites affecting gene expression. From left to right in each simulation, Simu is the variance explained by the known sites, Multi is the result fitting discovered eSNPs jointly, Uni is the result of summing the effects from sequential conditional modeling, and Single is the effect of the peak detected eSNP. The *y*-axis shows the proportion of variance explained. Scenarios are 4:0, all four minor alleles with effects in same direction; 3:1, one minor allele effect in the opposite direction; 2:2, two minor alleles on one direction and the other two in the opposite direction. eSNP, expression single nucleotide polymorphism.

Another way to consider the power of multisite detection is to ask how much of the variance explained by the four SNPs is captured by the discovered variants. Box and whisker plots in Figure 2 show that, under all three scenarios, on average 85–90% of the variance is captured, namely in these simulations ∼15–20% of the transcript abundance. Although effect sizes of all SNPs were drawn from the same distribution, the first discovered SNP (rightmost box in each panel) typically explains between one-third and one-half of the expected variance, suggesting that it often tags some of the effect of another site. Since the primary SNP captures on average more than two-thirds of the heritability at each locus in actual peripheral blood data (Lloyd-Jones *et al.* 2017), it is likely that secondary and tertiary SNP effects are, in reality, smaller than primary SNP effects. As expected, summation of the independent contributions from the sequential conditional models, or fitting all of the discovered variants simultaneously in a multivariable model, explains very similar proportions of the variance overall. Similar results are seen with two or three simulated causal variants (Supplemental Material, Figure S1 in File S1).

### Estimation of the proportion of secondary associations that are false positives

**Figure 3:**
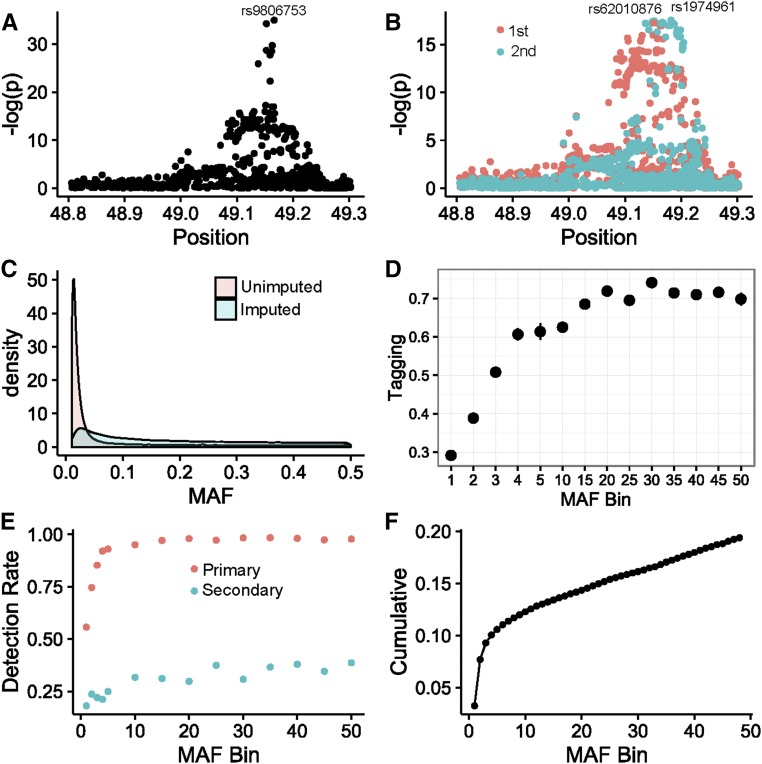
Proportion of false multiple eQTL detection due to unimputed variants. (A and B) Example showing how poor tagging can split one causal into two separate signals at the *SHC4* locus. (A) SNP rs9806753 (European-ancestry maf = 0.23) was simulated in CAGE to generated an eSNP effect, but removal of this variant and all SNPs within *r*^2^ > 0.5 from the analysis (B) results in the effect being captured by both a primary (rs62010876, maf = 0.10) and secondary (rs1974961, maf = 0.13) signals. (C) Empirical maf density distribution of 1.4 M unimputed 1000G (red) and 8.3 M imputed 1000G variants in CAGE (blue), demonstrating shift to lower frequencies for variants not tagged. (D) Tagging efficiency as a function of maf based in mean *r*^2^ for the strongest correlated SNP for 10,000 randomly selected variants across the frequency spectrum in the CAGE genotype dataset. (E) Corresponding signal detection rate at *P* < 10^−5^ for randomly assigned effect sizes, explaining between 2 and 8% of a simulated gene expression trait for primary (red) and secondary (blue) signals when the simulated variant is excluded from the analysis. (F) Cumulative proportion of sites expected to generate a false multiple eQTL detection, calculated as the sum of the false secondary signal detection rate weighted by the maf frequency in the indicated bins. Multiplication of this proportion by the number of unimputed SNPs in genic regions (14% of 1.4 M) and the actual proportion of SNPs that have effects (unlikely to be >1%) yields up to 400 possible false positive secondary associations. CAGE, Consortium for the Architecture of Gene Expression; eQTL, expression quantitative trait locus; eSNP, expression SNP; maf, minor allele frequency; SNP, single nucleotide polymorphism.

The detection of more association peaks than the number of simulated sites implies that some fraction of peaks are false positives that arise due to sampling artifacts in the presence of high LD, whereby an imperfectly tagged site is split into two or more spurious signals. An example is shown in Figure 3, A and B, contrasting the local Manhattan profiles for a single causal variant that splits into two associations when the peak SNP, rs9806753, is excluded from analysis. To explore the frequency with which this occurs, we conducted simulations assuming a single causal variant based on the genotypes measured in 1839 European-ancestry samples from the CAGE cohorts (see *Methods*). Randomly assigning causal effects resulted in the appearance of a secondary signal at *P* < 10^−5^, conditioned on the causal site, at 0.3% of the loci. This is approximately as expected given 8.3 million imputed SNPs at 22,000 loci, and is also the same as the false discovery rate of primary signals in the absence of any simulated causal locus; that is to say, our random expectation is for 0.3% of transcripts to have a false eQTL discovery at *P* < 10^−5^ in the CAGE dataset.

However, this ignores the possibility that the causal variants are not present in the imputed genotypes. Of the 9.7 million SNPs, indels, and CNV with maf > 0.01 in the European populations in the 1000G database, 1.9 million are not imputed in our CAGE samples. Figure 3C shows that the maf distribution for these variants is strongly shifted toward rarer alleles relative to the imputed SNPs, and is centered at a maf ∼0.02. Consistent with Yang *et al.* (2015), the average tagging efficiency (*r*^2^ value) of these SNPs is a function of maf, being >0.7 for maf > 0.05, but dropping to <0.5 for maf = 0.01, as seen in Figure 3D.

Since we cannot simulate effects at nonimputed SNPs, we approximated such alleles by randomly simulating a causal variant from the CAGE SNPs with the same frequency distribution, but excluding it from the analysis along with all variants that would tag it at the typical level observed for the nonimputed SNPs of the same maf. We then asked how often the effect is captured by multisite signals, as a function of residual tagging efficiency. We allowed for increased effect sizes with lower maf by simulating effects in the constant range of 2–10% of the variance explained. The proportion of such pseudounimputed SNPs that generate primary signals is reduced with lower maf, due in part to the smaller proportion of variance explained by the less common variants that partially tag them. For common variants, there is almost always a second site in high enough LD to capture most of the causal signal in the absence of genotypes at the causal variant, but rare variants are insufficiently tagged to generate a signal at all, 90% of the time.

In the presence of tagging SNPs with *r*^2^ > 0.5 to the “unobserved” causal variant, false secondary associations are observed ≤40% of the time. At the other end of the spectrum, rare variants (maf ∼0.01) that produce a primary signal at a tagging SNP with 0.1 < *r*^2^ < 0.3 also produce a secondary signal but less frequently. The blue curve in Figure 3E indicates the inferred fraction of unimputed variants that could induce secondary signals as a function of maf, and Figure 3F shows that the cumulative proportion of such spurious eQTL weighted by observed maf proportions approaches 20%. Approximately 14% of the 1.9 M unimputed variants are located within 200 kb of a gene, and assuming that 0.1% of these actually have an eQTL effect, this suggests the potential for ∼250 such effects.

These computations argue that up to 10% of the observed >2300 multisite associations reported by Lloyd-Jones *et al.* (2017) have the potential to be false signals driven by inefficient tagging of unimputed variants in CAGE. The proportion could be greater if the fraction of functional SNPs is higher, as suggested for example by Tewhey *et al.* (2016), who used a very sensitive MPR assay to implicate 3% of regulatory sites in 3642 eQTL regions (842/32,373 tests) as capable of modulating transcript abundance. However, the proportion of sites with detectable signals capable of explaining >2% of the variance is certainly lower, and 1 in 1000 (0.1%) is a reasonable estimate given that there are of the order of 1300 documented variants in the vicinity of each gene and no more than 30% of expressed genes have a secondary eQTL signal. Synthetic associations due to even rarer variants may be expected to generate split associations as well (Dickson *et al.* 2010; Zhu *et al.* 2012). Yang *et al.* (2015) found that ∼20% of the variance for height can be explained by SNPs with maf < 0.1, in part due to larger effect sizes of prevalent very rare SNPs, many of which are likely secondary associations. We also found that there is an excess of rare variants (maf < 0.01) influencing extremes of gene expression, also with a slightly larger distribution of effect sizes than common variants (Zhao *et al.* 2016). Too many unknown parameters need to be evaluated to give a good estimate of the number of false positive secondary associations due to synthetic effects of very rare alleles, but it may be another few percent.

### Effect of multisite modeling on accuracy of localization of associations

**Figure 4:**
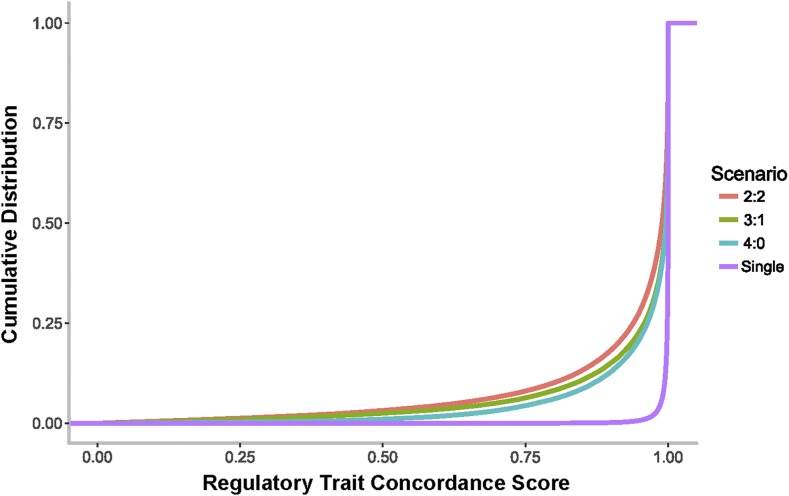
The single causal variant assumption biases fine mapping of causal variant locations. Each curve represents the cumulative probability distribution for RTC scores for the primary causal variants under a model with a single causal variant (purple), or with four causal variants under scenarios 4:0, 3:1, and 2:2 (blue, green, red curves, respectively). RTC scores close to one imply equivalence of the significance values of the eQTL and causal variant. eQTL, expression quantitative trait locus; RTC, Regulatory Trait Concordance.

A possibly more important measure of estimation bias is the location of the peak SNP relative to the causal site. The most straightforward measure of colocalization is the Regulatory Trait Concordance (RTC) score (Nica *et al.* 2010), which is intuitive and easily implemented on the scale of our simulations. It is essentially a ranking of the significance of the detected eQTL *P*-value relative to the casual site, RTC = (*N_SNPs_* − *Rank*_causal SNP_)/*N_SNPs_*, where a value of one indicates identity, and zero that the two sites are in the same locus but highly unlikely to be capturing the same signal. Figure 4 plots the cumulative frequency distribution for RTC scores for the primary eQTL signals relative to the largest effect causal variant in each simulation, contrasting the 4:0, 3:1, and 2:2 scenarios. For comparison, under a single variant model, RTC is always close to one as expected [note that, since we do not simulate the GWAS signal as well, these values are inflated relative to data where the identity of the actual causal variant is unknown (Nica *et al.* 2010)]. For 10% of simulations in the presence of multiple regulatory variants, the RTC score of the primary SNP drops below 0.9, again with greater tendency toward misestimation of the eQTL location in models with opposing effect directions of the minor alleles. This analysis confirms the results in Table 2, indicating that up to 15% of all detected SNPs are not in high LD (*r*^2^ > 0.8) with a simulated variant in the imputed panel of SNPs.

Localization of tertiary and quaternary signals is affected more strongly, but intriguingly, considering just associations within *r*^2^ > 0.8 of a simulated SNP, the secondary signal is slightly more likely to be the first or second ranked SNP for one of the causal variants than is the primary signal. This is true under all three scenarios, which have very similar profiles to that shown for the 3:1 scenario in Figure S2 in File S1 (since there is wide variance in the number of SNPs in each region, we simplified the analysis by reporting just the SNP ranks in this figure, rather than RTC). It should be noted that there is not strong concordance between the relative proportion of variance explained by the causal variants and whether they are the primary through quaternary association, since LD has a strong influence on detection power. Although the vast majority of discovered sites are within three or four SNPs of at least one of the four causal variants when they are in high LD with one of them, it cannot be concluded that the order of discovery corresponds to the true order of effect sizes.

### Joint fitting pairs of known causal variants accurately estimates effect sizes

Before addressing the accuracy of effect size estimation following stepwise conditional analysis, it is worth noting that, in the case where the identities of two causal variants are known *a priori*, joint fitting of the two SNPs in a single regression on transcript abundance always results in more accurate effect size estimates, given a large sample size. The bias in estimation due to LD between pairs of SNPs is a function of the two effect sizes (β_1_ and β_2_), the correlation between the SNPs (*r*), and the ratio of the square root of the product of their allele frequencies: Ê(β_1_)−β_1_ = *r*β_2_ √(*p*_1_(1−*p*_1_)/*p*_2_(1−*p*_2_)) (Yang *et al.* 2012). This is maximized for pairs of SNPs at the same frequency, increases with high LD, and can be either positive or negative depending on whether the signs of the minor allele effects are coupled or not. Figure S3 in File S1 provides a visual summary of the biases, compared with the effect of jointly fitting the two SNPs with a sample size of 2000, which uniformly improves the effect size estimates.

Three results deserve highlighting. First, for each combination of allele frequencies, increasing the allelic effect size results in more severe biases, in the most severe cases over- or underestimating the effects by as much as 50% of the variance explained. Second, the first picked SNP (with the larger effect size) has the greater deviation between the estimated and true effect size. This makes intuitive sense as the larger effect will generally be the first detected one and absorbs much of the effect of the other SNP, which will typically be underestimated in the conditional analysis, but to a lesser extent. If the deviation is computed simply on the uni-site unconditional values, the opposite result is obtained: the deviation is greatest for the smaller effect site. Third, the misestimation is greatest for lower allele frequencies, which is particularly noteworthy since most eQTL have maf in the range of 0.1–0.3.

We also considered the power to detect joint effects in the presence of LD. As the *P* value cutoff for detection becomes more stringent, the bias in estimation becomes more severe, since the first picked SNP absorbs the effect of both alleles into the same estimate, leaving the statistical power of the other allele, conditioned upon the first one, close to zero. With a sample size of 2000 and intermediate LD, when both alleles are modeled jointly, power to detect both effects remains high across the plausible parameter space once the effect size exceeds 5% variance explained, and the estimation for the β value is still accurate. Downsampling suggests that in order to estimate effects within 0.1 sdu, for pairs of variants with LD *r*^2^ ∼ 0.9, each explaining 10% of the variance (namely having effect sizes of at least 0.5 sdu), a sample size of at least 900 is required.

Consequently, most small sample eQTL studies will fail to resolve linked sites into two effects. These results indicate how the typical assumption that an eQTL effect is due to a single variant in a set of credible SNPs in high LD is potentially highly biased. Similar conclusions apply to the situation where two SNPs operate in opposite directions, with the additional dilemma that they will not be detected at all and consequently strongly underestimate the regulatory variance at a locus.

### Misestimation of allelic effects sizes by sequential conditional analysis

**Figure 5:**
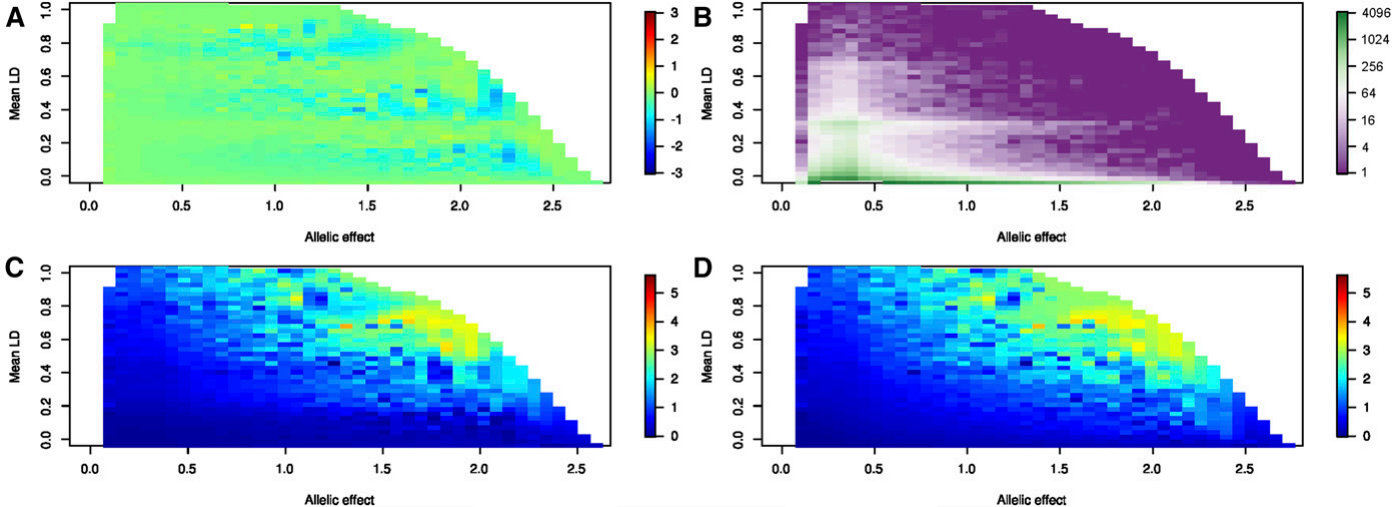
Biases in effect size estimation from conditional and joint analysis. All panels refer to 500,000 simulated data points where effect sizes were sampled from a uniform distribution to explain from 2% to 10% of the expression at a locus for each of 4 SNPs picked at random from 400kb intervals of the CAGE genotype data. Panel (A) compares estimates from joint and conditional modeling, as a heatmap of the average difference in panels C and D, where yellow indicates that joint modeling produces a larger estimated effect size, and blue a lower estimate with three bands of negative values indicating greater bias in the conditional estimates. Panel B shows the density distribution on the log2 scale of the number of simulations with alleles for each pixel with the indicated β (in standard deviation units, sdu) on the x-axis, and average LD with the other 3 sites at the locus on the y-axis. Panels C and D show the average absolute value of the deviation between the observed and known effect size for sites under the multi-site model where all discovered sites are fit jointly (C) or from single site estimates after each step of sequential conditional analysis (D), for the 4:0 scenario where all minor alleles have effects in the same direction. See Figure S4 for the equivalent panels for the 3:1 and 2:2 scenarios.

Even though the sequential conditional and multisite models capture essentially equivalent proportions of the variance tallied across sites, biases in estimation of individual site effects ought to be reduced by the multisite modeling. To quantify this difference, we computed the deviation between the observed and true simulated effect sizes (β in sdu) for each discovered peak located within *r*^2^ > 0.8 of an independent causal variant, and evaluated the absolute value of these deviations as a function of the mean LD between the causal variant SNP and the other three causal variants in the model. Figure 5, A and B show the average absolute value of the deviation for SNPs with the indicated true effect size and LD, for the sequential conditional estimates, and for multivariable models fitting all discovered variants jointly, respectively. The figure shows results for the 4:0 scenario where all minor alleles operate in the same direction. The scale from dark blue to yellow indicates misestimation of effect sizes ranging from less than 0.5 sdu to more than three units, where each pixel is averaged over the number of simulations with the indicated allele effect sizes and average LD in Figure 5C.

Several results are noteworthy. First, for causal variants in low LD, as expected, neither model results in appreciable estimate bias, but once the average LD rises above 0.5, effects can be misestimated by more than the effect size. For example, for β = 0.5, the absolute value of the difference between the observed and true effect is typically between 1 and 2 sdu, which depending on the allele frequency may correspond to at least 2% of the total gene expression variance. Second, for large effect alleles, the misestimation is appreciable even at intermediate levels of LD, and it is not unusual for estimates to be off by as much as 4 sdu under either model.

Third, overall, the multisite modeling corrects some of the sequential conditional analysis bias. The difference in performance of the two estimation procedures is shown in Figure 5D, a plot of the average multisite estimate minus the average sequential conditional estimate. Most values are pale green, indicating close similarity of the estimates, but bluish-tinged bands imply that the multisite model gives a better approximation to the true effect size for LD centered ∼0.1, 0.4, and 0.8. Misestimation without multivariate estimation can be twice as severe for very large effect alleles, although these only account for a very small fraction of all simulated alleles.

Similar trends are seen for the 3:1 scenario, where one of the minor alleles operated in the opposite direction to the other three, as well as in the 2:2 scenario (Figure S5 in File S1), and simulations with just two or three causal variants yield similar conclusions (Figure S6 in File S1). The advantage of joint modeling is reduced in the presence of opposing allelic effects, but still prevalent in the region with high allelic effect size and low LD; and, as noted above, a large proportion of causal sites are not discovered in the 2:2 scenario, so are not included in the estimation.

In summary, stepwise conditional eQTL discovery is expected to discover between 70 and 80% of eQTL within realistic effect size ranges typical of those reported in the literature. Once discovered, multi-locus estimation of effect sizes provides slightly more accurate estimates than the estimates from sequential conditional models, but for large effect alleles in high LD the corrections can be substantial.

### Bayesian modeling only slightly improves mapping of multisite associations

**Figure 6:**
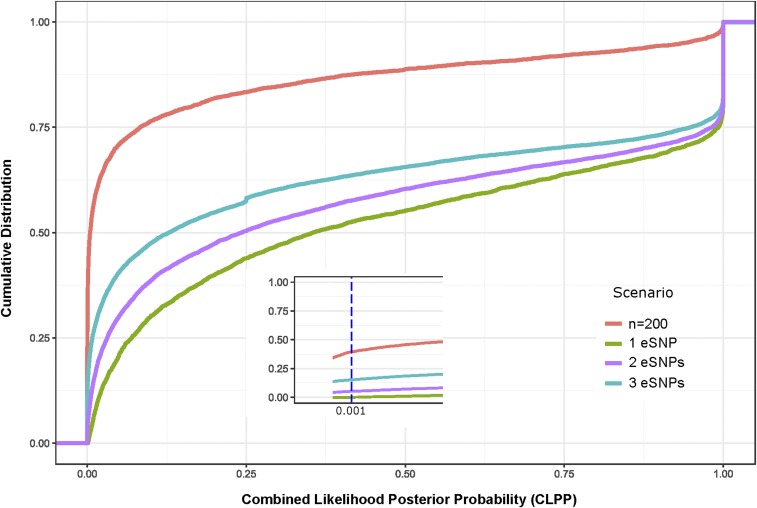
Colocalization with eCAVIAR in the presence of multiple regulatory sites. Cumulative Distribution Functions summarize CLPP scores for 4000 simulations each with one, two, or three assigned causal sites within a contiguous block of 100 SNPs. Green, purple, and teal curves show progressive degradation of evidence as the number of modeled causal variants increases, for simulations with 1835 subjects. The red curve shows that more than half of the simulations with just 200 subjects for the eQTL component have CLPP < 0.01, and just one-quarter >0.1, compared with one quarter >0.98 with 1835 subjects. CLPP, Combined Likelihood Posterior Probabilities; eQTL, expression quantitative trait locus; SNP, single nucleotide polymorphism.

Recently, a number of Bayesian approaches have been introduced that are designed to improve fine mapping of eQTL effects (Giambartolomei *et al.* 2014, Zhou *et al*. 2013). One of these is eCAVIAR (Hormozdiari *et al.* 2016), which reports a CLPP based on the combined likelihood that a variant influences both the abundance of a transcript and a phenotype given the LD structure at a locus. The authors proposed a CLPP cutoff of 0.001 (for example, a posterior probability of 0.1 for the eQTL and 0.01 for a disease association), which corresponds in our simulations (Table 3) to discovery of 80.7% of single variants sampled at random from contiguous blocks of 100% SNPs in the CAGE European-ancestry cohort genotype data. The computational burden of evaluating all possible four site combinations is too large for this model to be applied in genome-wide scans. Instead, we performed 4000 simulations of 1835 individuals in the presence of two or three regulatory variants, as well as a normally distributed phenotype, and evaluated the CLPP distributions. In the case of two causal variants, just 94.7% generated CLPP > 0.001, and for three causal variants, 84.9%. Figure 6 shows the cumulative distribution functions of the CLPP scores as a function of the number of causal variants, clearly documenting the trend for reduced confidence in joint localization as the degree of multisite regulation increases. Similar trends were seen with a more conservative CLPP cutoff of 0.01, confirming that interference among tightly linked sites reduces the power to detect independent causal variants. The upper red curve also indicates that the power to detect colocalization is greatly reduced with sample sizes of just 200 for the eQTL sampling: in fact, just 60% of simulations with a single causal site yielded a CLPP > 0.001.

**Table 3:** Effect of multisite regulation on colocalization of eQTL with eCAVIAR

Number of Sites	Number of Causal Variants Simulated (*n* = 1835)			*n* = 200
	1	2	3	1
Tested	3960	7880	11,830	3999
CLPP > 0.001	3959	7466	10,038	2426
Proportion	100%	94.7%	84.9%	60.7%

CLPP, Combined Likelihood Posterior Probabilities.

Since eCAVIAR is not designed to cover intervals encompassing all of the regulatory regions of a typical gene and hence is not directly comparable with the stepwise conditional regression, we also evaluated the DAP algorithm (Wen *et al.* 2016). DAP is designed to identify independent credible intervals and report candidate SNPs across a locus, incorporating priors that weight likely functional or evolutionary evidence. Using the adaptive DAP procedure, which does not make any prior assumptions about the number of causal variants at a locus, we performed 130 simulations of four variants with effect sizes drawn as before to explain between 2 and 10% of the variance, all operating in the same direction. The average number of detected variants was 3.76, which included 3.52 of the four simulated sites (88%) in the candidate list. Due to different statistical thresholds, it is difficult to compare this result with the stepwise conditional model, but it appears to be an improvement on the 80% reported in Table 2.

**Figure 7:**
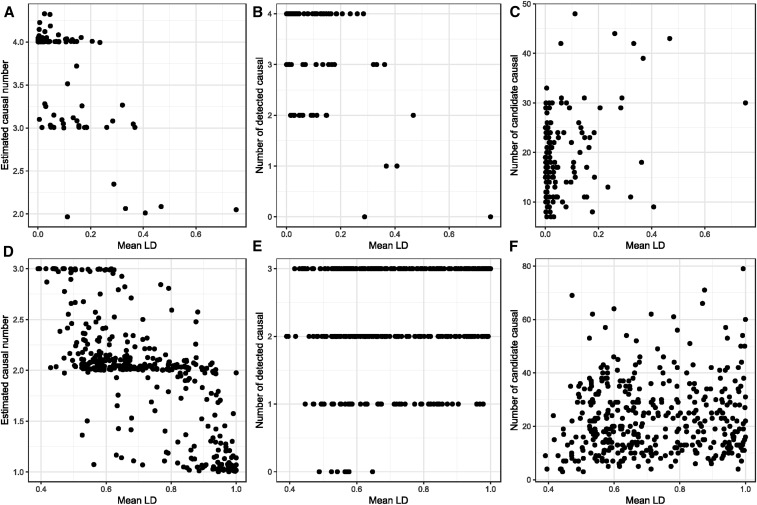
Fine mapping with DAP in the presence of multiple regulatory sites. (A–C) Results for simulations with four causal sites drawn at random from 200 kb upstream and downstream of each gene. (D–E) Results for simulations with three causal sites drawn from 100 continuous SNPs, each in LD with *r*^2^ > 0.3. (A and D) show the estimated number of sites as a function of the mean LD between the sites, showing that as LD increases, detection of independent intervals decreases. (B and E) show the number of modeled (true) causal sites in the candidate lists, as a function of mean LD, which in this case increases for high LD. (C and F) show that the number of candidate sites increases with high LD, sometimes with 20 or more candidates defining a credible set for each true site. DAP, Deterministic Approximation of Posteriors; LD, linkage disequilibrium.

As expected, DAP fails to detect true causal variants in the presence of high LD. Figure 7A shows the dependency of the number of discovered variants on the mean LD between the four simulated sites in each simulation, while Figure 7B shows how many of the true causal variants are detected. Notably, if all four variants are in a single block of high LD, no sites are detected since the posterior probabilities are dispersed across all of the variants. Across the full extent of up to 500 kb at most loci there are usually multiple LD blocks, so DAP, like stepwise conditional modeling, is quite efficient at detecting independent credible intervals. However, it consistently overreports the number of candidate variants and Figure 7C shows that this number also increases with LD. To confirm that DAP is still able to resolve multiple causal variants in the presence of high LD, we also ran 400 simulations with the constraint that three sites must be within *r*^2^ > 0.3, using the DAP-k algorithm with *k* = 3 (assuming three sites), showing the results in Figure 7, D–F. In this case, the number of detected independent associations dropped to 1.95, namely 65% of the simulated number. Although 79% of the simulated variants were among the candidate lists, these can become very large with a ratio of ten-to-one candidates for each true causal variant. Stepwise conditional analyses on the same simulations with a cutoff of *P* < 10^−5^ discovered on average 1.56 (52%) of the three simulated effects and included 2.29 (76%) of the sites within the credible interval. Consequently, DAP does appear to improve performance, at the cost of a considerably higher computational burden.

## Discussion

Studies of the genetic regulation of gene expression are making a meaningful contribution to the interpretation of GWAS results, as they provide functional insight into the nature of the causal genes. However, efforts to fine map causal variants are complicated by the limits of statistical resolution as it is not uncommon for tens, if not hundreds, of polymorphisms in a credible set to have similar statistical support (Gaulton *et al.* 2015; Kichaev and Pasaniuc 2015). Inclusion of experimental evidence from epigenetic marks or signatures of evolutionary conservation into scores such as CADD (Kircher *et al.* 2014), CATO (Maurano *et al.* 2015), and LINSIGHT (Huang *et al.* 2017) promises to improve resolution, as do methods such as RTC (Nica *et al.* 2010) and PICS (Farh *et al.* 2015), which prioritize variants based on the structure of LD at a locus. In general, these approaches assume parsimony, namely that there is a single variant that is responsible for the major GWAS or eQTL signature. Although it has become increasingly clear that many loci harbor multiple independent regulatory variants, we argue here that if the parsimony assumption is relaxed and it is assumed that multiple sites in strong LD commonly account for a signature that is compounded into a single significant association, then the estimates from sequential conditional analysis can be highly biased. To summarize, we find that >5% of primary sites and more than one-fifth of all causal sites are unlikely to be tagged at all; that in the presence of multisite regulation at least 15% of all mapped sites are not in strong LD with any of the multiple imputed causal variants at a locus; and that another 10% of the associations are plausibly due to splitting of the signal due to an unimputed site. Taken together with increasing evidence that up to a third of all eGenes have two or more independent eSNPS (Gusev *et al.* 2014; Wen *et al.* 2015; Lloyd-Jones *et al.* 2017), these results suggest that at least 5% and perhaps as many as a one-quarter of mapped credible intervals may not include the actual causal variant.

Theory and simulation both indicate that if two linked sites both influence a trait, including gene expression, then multisite models will uniformly outperform sequential uni-site ones with regard to estimation of the true effect size. When the identities of the variants are known, sample sizes of several thousand individuals are sufficient to jointly estimate their effects with high accuracy even in the presence of high levels of LD with *r*^2^ up to, or even exceeding, 0.9. The problem is that the identities of the variants are generally not known, and there are no established methods for comprehensive screening transcriptome-wide for localization of multi-locus local eQTL effects. Two exhaustive search algorithms, PAINTOR (Kichaev *et al.* 2014) and CAVIARBF (Chen *et al.* 2015), hold promise for detailed dissection of multisite models at individual loci, and a Bayesian shotgun stochastic search algorithm, FINEMAP (Benner *et al.* 2016), has recently been proposed for rapid maximum likelihood estimation of multi-SNP contributions. Here, we show by simulation that DAP (Wen *et al.* 2016) does indeed improve on sequential conditional analysis for localization of multiple linked causal variants, but that in regions of high LD its performance remains constrained. Given the computational burden, it may be more efficient to use stepwise modeling, perhaps supplemented with a lasso regression method, to map independent sites in low LD, and then concentrate on each credible interval with these Bayesian fine mapping tools. It should also be recognized that single site effects may sometimes be artificially split into two or more linked contributions under each of these strategies.

We also estimated the bias in the estimates from conditional analysis, by fitting multi-locus linear models to all of the discovered eSNPs at each locus. This revealed only modest improvements in accuracy for most of the discovered sites, but the modesty is in part an artifact of the discovery bias introduced by the sequential conditional process. Our simulations assuming two to four effective sites per locus across a wide and representative range of LD, show that in a sample size of 2000, in general no more than 85% of the simulated causal sites are tagged by discovered associations that explain typically observed magnitudes of effect and would almost always be detected if a single site explained the variance. Similarly, Lloyd Jones *et al.* (2017) estimate that, in the CAGE dataset, on average between 50 and 75% of the heritability due to locally acting regulatory polymorphism can be attributed to discovered variants. Multisite modeling readjusts the remaining estimates typically by between 0.1 and 0.5 sdu, which, depending on the allele frequency, accounts for between 2 and 5% of the variance explained, and only rarely >10%.

However, any variants with effect sizes >1 sdu, and whose average *r*^2^ with the other three SNPs is >0.9, will be misestimated in both the single site and joint models, typically by 1.5 sdu or more. The misestimation is on average the greatest where all of the effects are in the same direction, but is consistently observed also in the presence of associations with alternate signs. Unavoidably, the sequential conditional estimates of eQTL effects are actually highly biased for a considerable proportion of variants. Although this does not impact the total amount of variance explained by the discovered variants, it is likely to greatly impact fine mapping efforts, particularly where two or more effects are collapsed into one site in a credible interval.

While large datasets have very good power for detection of complex regulatory contributions for individual genes, there are a host of technical and statistical reasons why fine mapping of causal variants remains a challenge. There are two immediate strong implications of these results. One is that even though the majority of identified eSNPs are expected to map to credible intervals that include the causal variant (Gusev *et al.* 2014; Finucane *et al.* 2015), there will also be many instances where incongruence between the statistical interval and chromatin or other functional evidence (Huang *et al.* 2015) is to be expected. The causal variant may simply be poorly mapped due to interference among linked functional sites. This effect may also influence the fine mapping of pleiotropic associations (Fortune *et al.* 2015). The second implication of the high frequency of multisite regulation is to emphasize caution in using univariate statistical support for an eSNP effect as sufficient evidence that an association between a SNP and a trait is evidence for causation. At a minimum, it is imperative that the full spectrum of eSNP effects across the locus be evaluated to confirm that the site is not simply in LD with higher likelihood eSNPs that are not themselves associated with the trait. Experimental validation of individual sites seems warranted in situations where establishment of the identity of the causal variant(s) is desired.

## Supplementary Material

Supplemental material is available online at www.g3journal.org/lookup/suppl/doi:10.1534/g3.117.043752/-/DC1.
