# Supplementary material for: Constraints on eQTL Fine Mapping in the Presence of Multisite Local Regulation of Gene Expression
Source: G3 (Bethesda). 2017 Jun 8;7(8):2533–44. doi: 10.1534/g3.117.043752 (PMC5555460; doi:10.1534/g3.117.043752)
Supplement: Supplementary file 1 [file 2533File001.pdf]

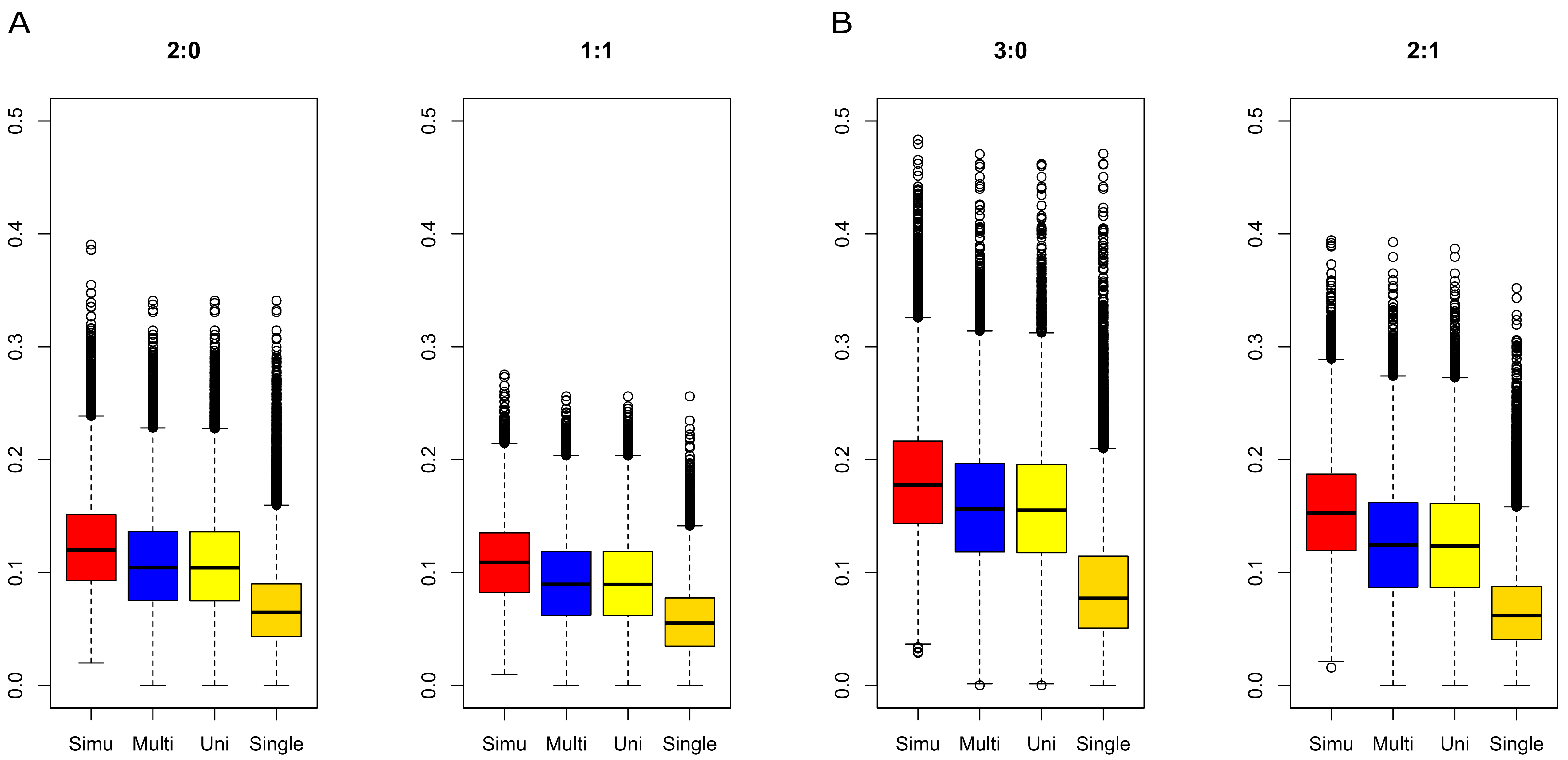

Figure S1. Effect size estimation bias under the three scenarios with 4:0, 3:1, or 2:2 directions of eSNP effects. Panels from left to right in each row show the average absolute value of the difference between the estimated multisite β and true β, the absolute value of the difference between the estimated sequential conditional β and true β, and the average difference between these values. Each scenario includes 500,000 simulations as in Figure 2.

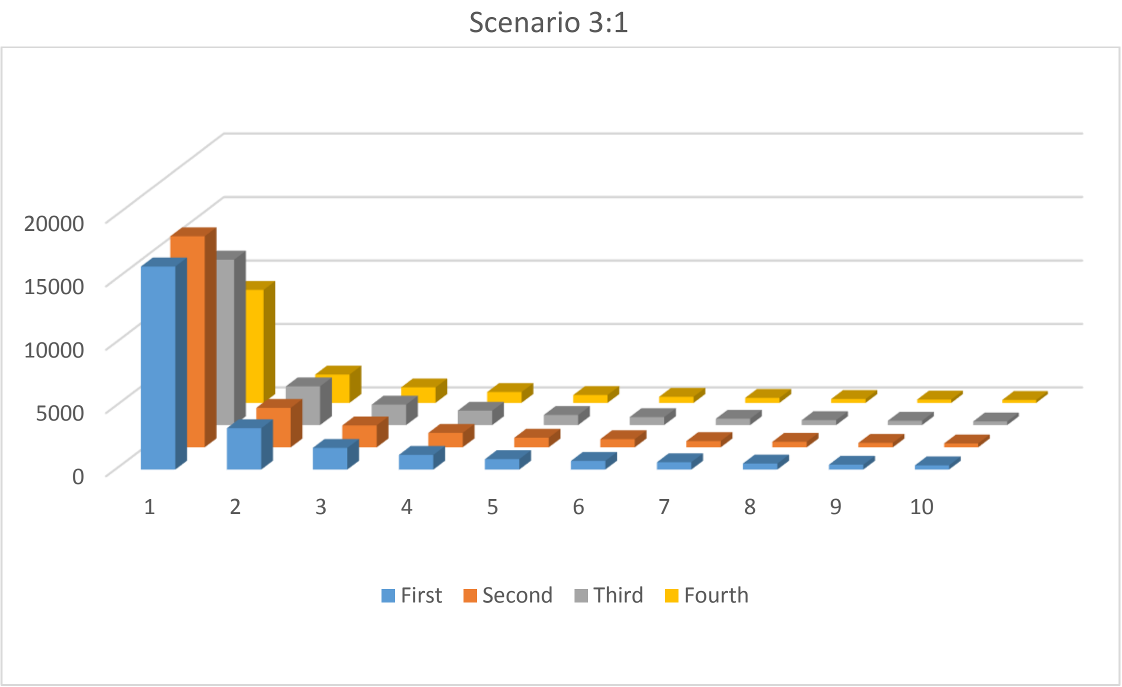

Figure S2. Signal ranks of simulated causal variants. The number of simulations in which a discovered variant was the indicated rank (left to right) for the first through fourth discovered variant (front to back). Rank refers to the number of SNPs with a smaller p-value than the modeled causal variant, where 1 implies the causal and discovered are the same SNP, 2 that one other SNP in the LD region had a smaller p-value, and so forth. Only cases where the discovered variant was within r2 > 0.8 of the causal variant are shown.

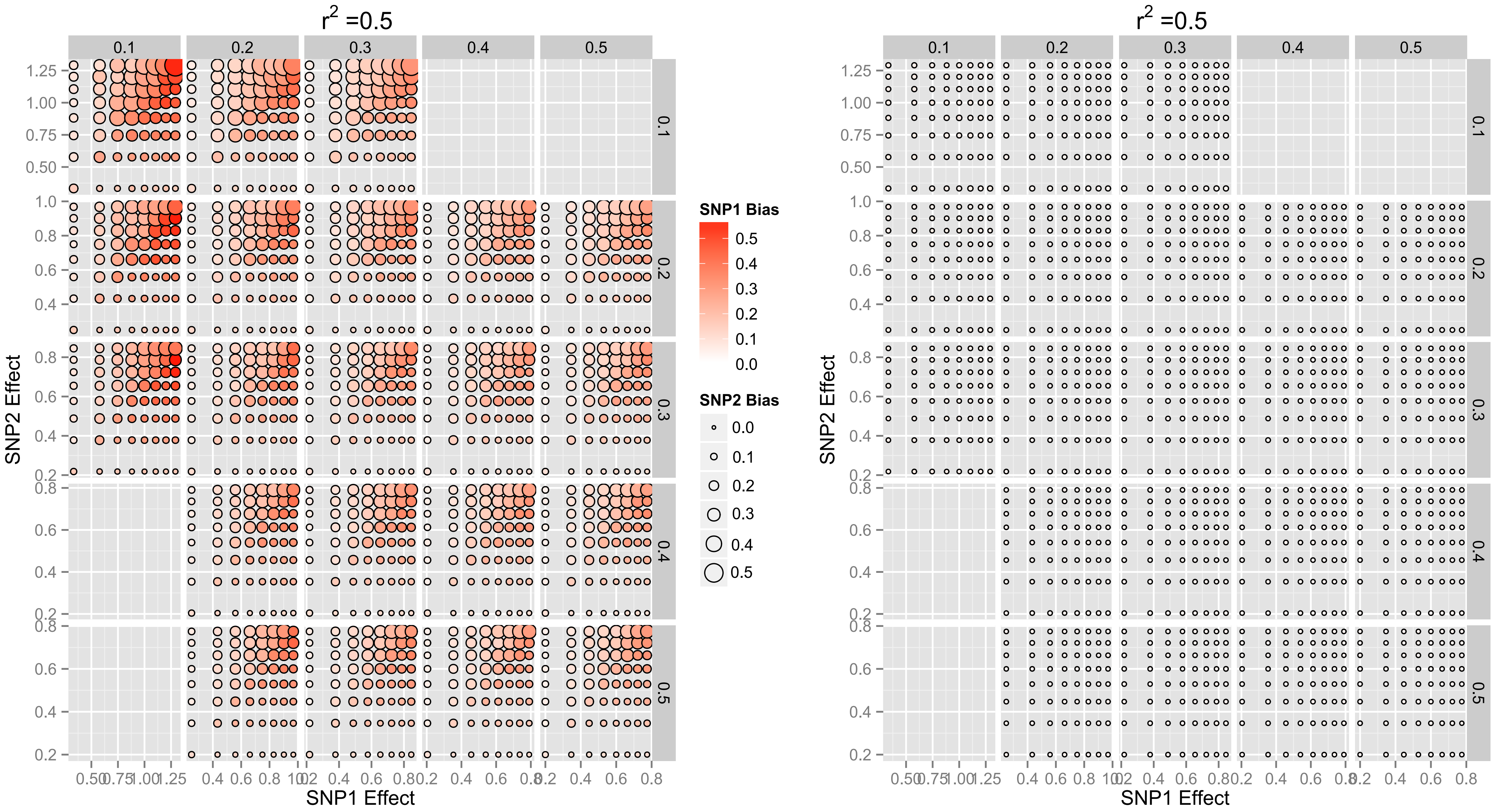

Figure S3A. Simulation of the influence of minor allele frequency and β on allelic effect size estimation. Left side, sequential conditional modeling; right side joint multi-site modeling, with n= 2,000 and LD r2 = 0.5. The 25 sectors on each panel show results for combinations of two alleles with maf from 0.1 to 0.5 (left to right, top to bottom), within which each circle represents the average of 100 replicate simulations for allelic effects explaining from 2% to 30% of the expression variance (left to right, bottom to top). Circle size and color is proportional to the absolute value of the deviation between the estimated and actual effect size β in standard deviation units. Transition from white to red represents greater deviation for allele 1; larger circles represent greater deviation from the simulated β for allele 2.

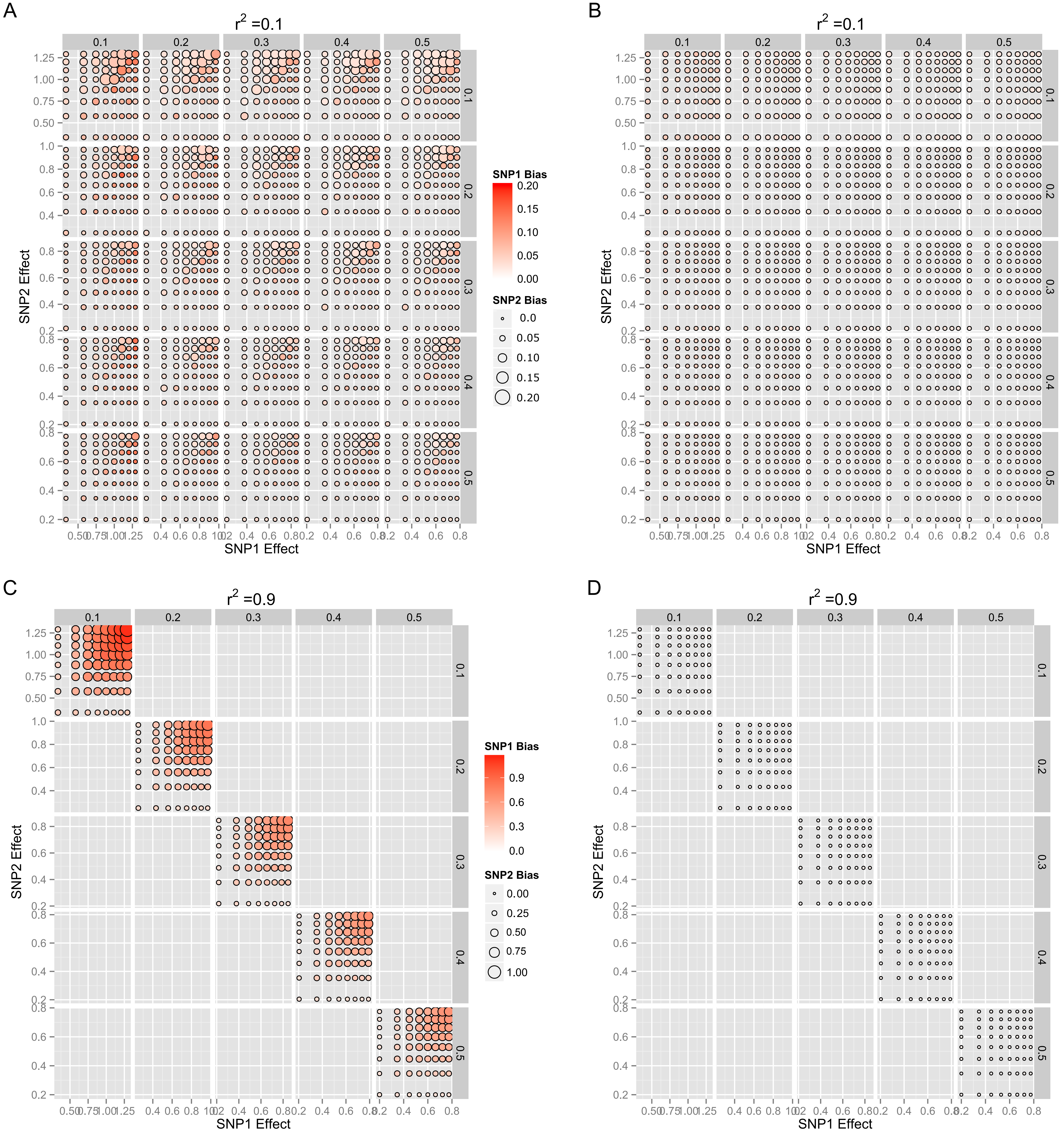

Figure S3B. Simulation of the influence of minor allele frequency and β on allelic effect size estimation. As in Figure S3A, but Top LD r2 = 0.1, and Bottom LD r2 = 0.9. Empty fields arise because the indicated level of LD is not possible for the corresponding allele frequencies. Results at varying levels of LD show that sequential univariate biases are particularly strong in the presence of high LD (bottom left), but less pronounced for low LD (midddle left).

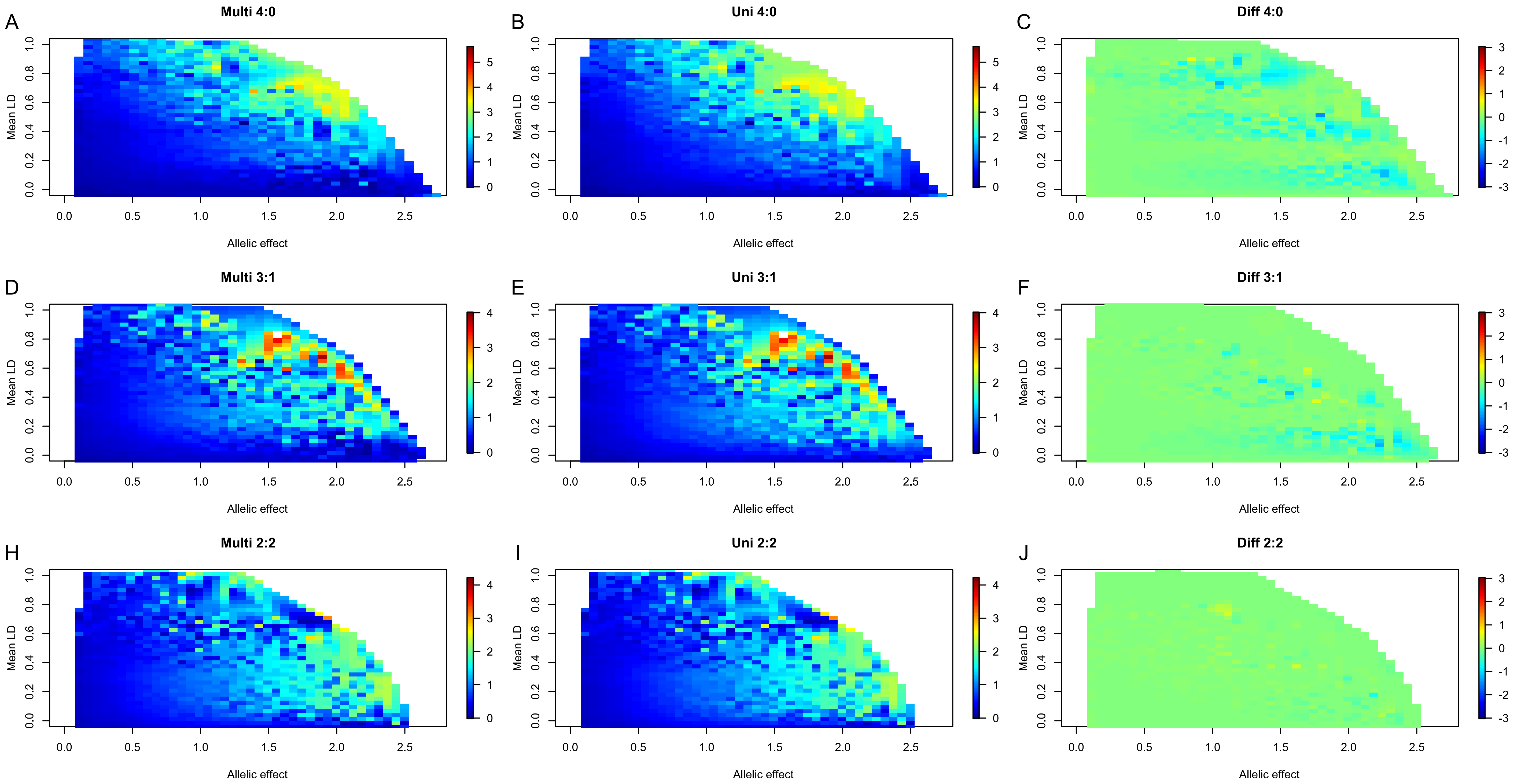

Figure S4. Effect size estimation bias under the three scenarios with 4 causal variants. Each scenario includes 90,000 simulations and panels from left to right in each row show the average absolute value of the difference between the estimated multisite β and true β, the absolute value of the difference between the estimated sequential conditional β and true β, and the average difference between these values. Top row is results for four sites where the minor alleles have the same sign of effect, bottom row for two sites each with the same sign, middle row a mixture of three in one direction and one in the other.

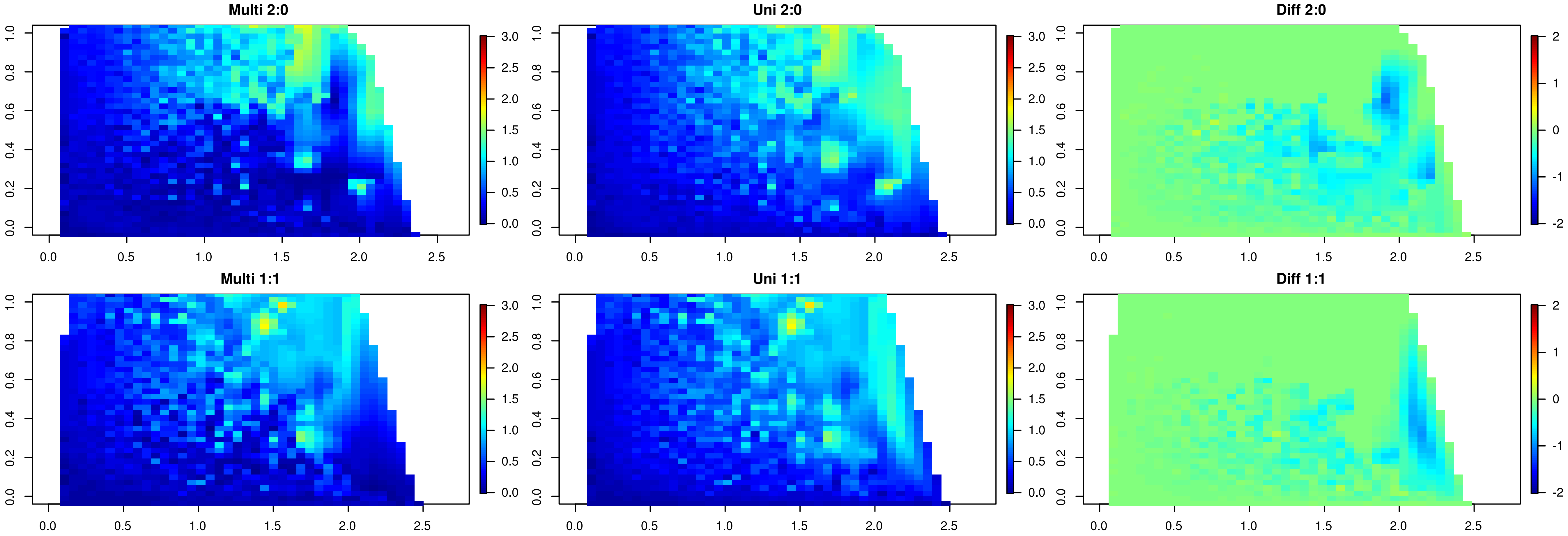

Figure S5A. Effect size estimation bias under the two scenarios with 2 causal variants. Each scenario includes 90,000 simulations and panels from left to right in each row show the average absolute value of the difference between the estimated multisite β and true β, the absolute value of the difference between the estimated sequential conditional β and true β, and the average difference between these values. Top row is results for two sites where the minor alleles have the same sign of effect, bottom row for two sites with the same sign.

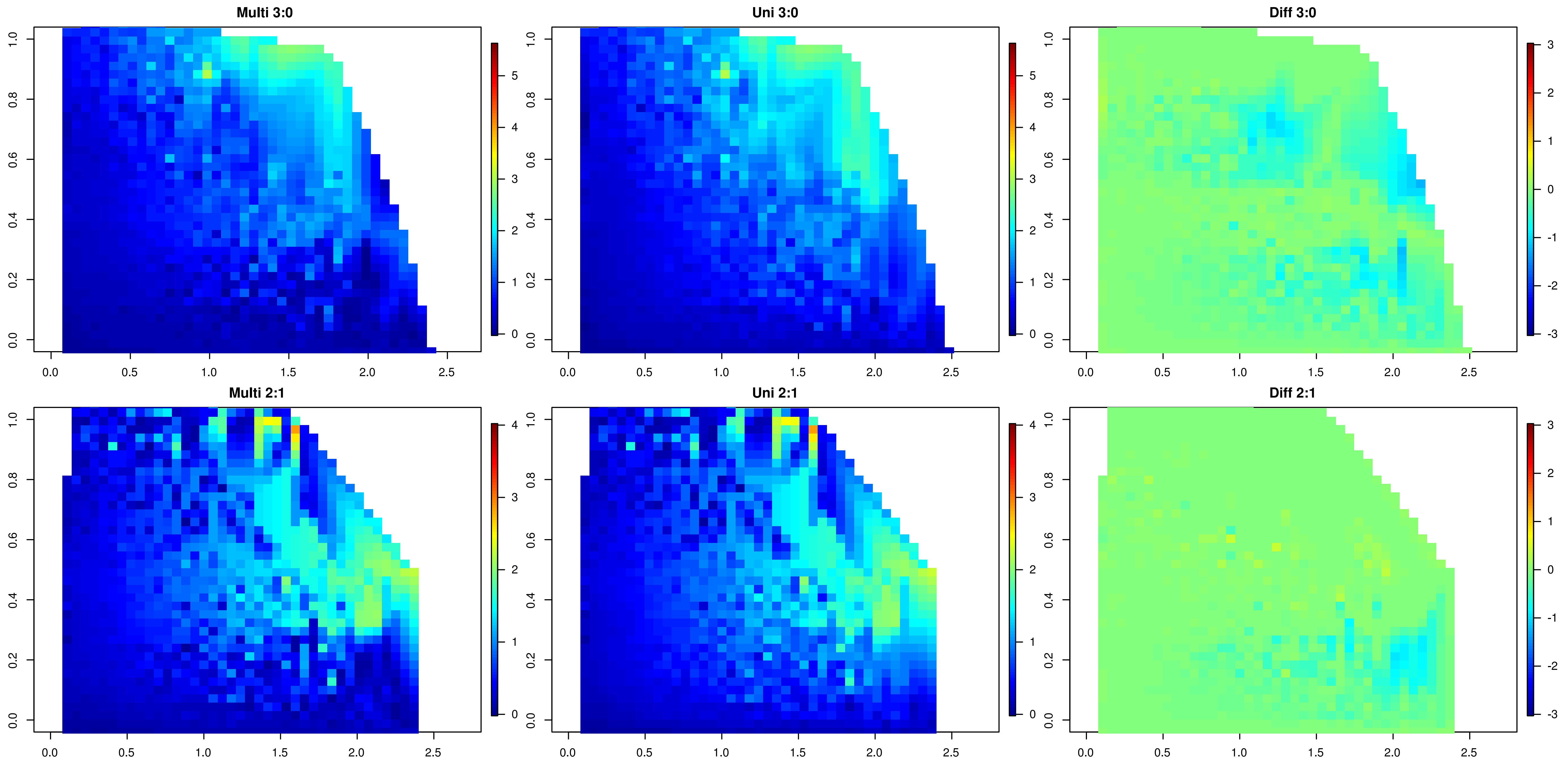

Figure S5B. Effect size estimation bias under the two scenarios with 3 causal variants. Each scenario includes 90,000 simulations and panels from left to right in each row show the average absolute value of the difference between the estimated multisite β and true β, the absolute value of the difference between the estimated sequential conditional β and true β, and the average difference between these values. Top row is for three sites, all with the same sign, and bottom row is one site operating in the opposite direction to the other two.
